# Climate influences the gut eukaryome of wild rodents in the Great Rift Valley of Jordan

**DOI:** 10.1186/s13071-024-06451-x

**Published:** 2024-08-23

**Authors:** Sanaz Khadem, David Berry, Enas Al-khlifeh

**Affiliations:** 1 Centre for Microbiology and Environmental Systems Science, Department of Microbiology and Ecosystem Science, Division of Microbial Ecology, University of Vienna, Vienna, Austria; 2 Joint Microbiome Facility of the Medical University of Vienna and the University of Vienna, Vienna, Austria; 3 Laboratory of Immunology, Department of Medical Laboratory Science, Al-Balqa Applied University, Al-Salt, Jordan

**Keywords:** Eukaryome, Metabarcoding, Spatial variability, Bioclimatic zone, *Musmusculusdomesticus*, *Acomyscahirinus*

## Abstract

**Background:**

The mammalian gut microbiome includes a community of eukaryotes with significant taxonomic and functional diversity termed the eukaryome. The molecular analysis of eukaryotic diversity in microbiomes of wild mammals is still in its early stages due to the recent emergence of interest in this field. This study aimed to fill this knowledge gap by collecting data on eukaryotic species found in the intestines of wild rodents. Because little is known about the influence of climate on the gut eukaryome, we compared the composition of the gut eukaryotes in two rodent species, *Mus musculus domesticus* and *Acomys cahirinus*, which inhabit a transect crossing a temperate and tropical zone on the Jordanian side of the Great Rift Valley (GRV).

**Methods:**

We used high-throughput amplicon sequencing targeting the 18S rRNA gene in fecal samples from rodents to identify eukaryotic organisms, their relative abundance, and their potential for pathogenicity.

**Results:**

Nematodes and protozoa were the most prevalent species in the eukaryome communities, whereas fungi made up 6.5% of the total. Sixty percent of the eukaryotic ASVs belonged to taxa that included known pathogens. Eighty percent of the rodents were infected with pinworms, specifically *Syphacia obvelata*. Eukaryotic species diversity differed significantly between bioclimatic zones (*p* = 0.001). *Nippostrongylus brasiliensis* and *Aspiculuris tetraptera* were found to be present exclusively in the Sudanian zone rodents. This area has not reported any cases of *Trichuris* infections. Yet, *Capillaria* infestations were unique to the Mediterranean region, while *Trichuris vulpis* infestations were also prevalent in the Mediterranean and Irano-Turanian regions.

**Conclusions:**

This study highlights the importance of considering host species diversity and environmental factors when studying eukaryome composition in wild mammals. These data will be valuable as a reference to eukaryome study.

**Graphical Abstract:**

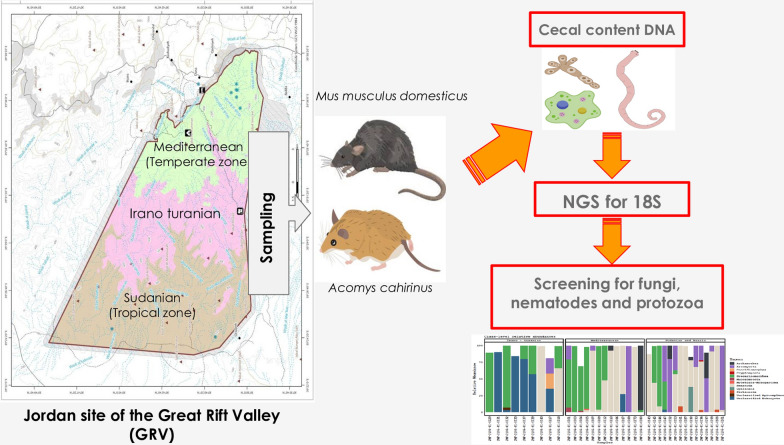

**Supplementary Information:**

The online version contains supplementary material available at 10.1186/s13071-024-06451-x.

## Background

To classify the full range of nucleated organisms, including helminths, protozoa, and fungi, that live in the intestines of different animals, parasitologists have adopted the term "eukaryome" [1]. Previous research on the gut microbiome has mostly focused on characterizing the bacterial composition or certain interactions between bacteria and eukaryotic organisms. This could be attributed to the fact that most members of the studied gut microbiome are prokaryotic, while eukaryotic communities make up only 2–5% of the total [2]. Overall, the diversity of the gut ecosystem can have varying effects on hosts due to the communication and potential increase in richness among intestinal organisms. The relationship between increased microbiome diversity and helminth or *Entamoeba* load in the gut [3, 4] lends support to this. Thus, microbiome investigations must incorporate eukaryome screening.

Different types of symbiotic relationships can be found in the gut eukaryome. These include parasitic, commensal, and mutualistic symbioses. Although the harmful functions of parasites and fungi are usually mentioned, the harmless activities of host-associated eukaryotic organisms have recently received increasing attention. Like the microbial communities of herbivores, protozoa may have a direct or indirect role in digestion [5]. Furthermore, a new study on omnivores has shown that carbohydrate content in the diet correlates with the abundance of yeast and fungi in the gut, which could indicate that fungi play a role in the breakdown of carbohydrates in the colon [6]. Research on humans has revealed that the gut protists *Blastocystis* and *Dientamoeba fragilis* are present in relatively high numbers in healthy individuals [7]. There is a strong correlation between the gut eukaryome and host immune system. One example is the anti-inflammatory effects of several helminth species, such as hookworms and *Trichuris* sp., which are known to selectively activate the type 2 immune response [8]. Alternatively, fungi can influence the immune system through their cell wall components or by secreting enzymes and poisonous compounds [9].

There are still a limited number of studies on the eukaryome of animals. However, studying entire host-symbiont communities is gaining importance in several fields, including mycology and parasitology. By sequencing the SSU rRNA genes at the metagenomics and DNA metabarcoding scales, it was found that the eukaryome is more diverse than usual morphological methods would suggest [10]. This approach has recently helped to clarify the phylogeny of the host eukaryome [11]. However, most studies have only examined a single group of eukaryotic organisms. For instance, metabarcoding has been used to study nematodes in wild rats and mice [12, 13]. Similarly, 18S rRNA gene metabarcoding has been applied to study protozoa in human and animal host feces [14–16]. However, there is less research available about the gut eukaryome of wild mammals including but not limited to the eukaryome of Spotted hyenas by Heitlinger et al. (2017) [17], bats by Li et al. (2018) [18], nonhuman primate by Mann et al. (2020) [19], and the *Apodemus agrarius* by Kim et al. (2022) [20].

Wild rodents constitute approximately 40% of all mammalian species globally and contribute significantly to the biodiversity of the Eastern Mediterranean [21]. Moreover, they are widespread in all types of landscapes and have distinct life histories. As a result, rodents have become an essential component of the interaction between humans and wildlife. Research on the factors influencing the eukaryotic makeup is best conducted using rodents. They offer a unique perspective for studying eukaryotes in the wild. Direct comparisons between laboratory and wild mouse eukaryome research are made possible by the use of *Mus musculus domesticus* as a key laboratory animal in many intestinal parasite investigations [22, 23]. However, to date, few research studies have been done on the wild rodent eukaryome, and much less has been done to identify the factors that influence its composition.

Across Jordan's four distinct biogeographical zones, wild rodent distributions mirror the worldwide pattern of biodiversity [24]. Some rodents, including *A. cahirinus* and *Mus musculus*, have a wider range of habitats than previously thought [25]. In most cases, they serve as generalist hosts. They are often thought of as major carriers of vector-borne diseases. The spread and incidence of infectious diseases are greatly affected by this phenomenon. Generally, parasites tend to be enriched in the tropics, and parasitic diseases are common in these areas [26]. Because a wide variety of parasitic diseases can thrive in warm, tropical, and subtropical environments where species diversity is encouraged, rising temperatures have a direct impact on the prevalence of many organisms since many eukaryotic parasites have a developmental baseline that is reliant on temperature, either inside their host or in the environment. Among the few studies that have characterized the eukaryome of wild mammals, comparisons between host species have revealed that the intestinal eukaryome compositions of various host species vary significantly [17–20]. The eukaryome composition of the Spotted hyena (*Crocuta crocuta*) was shown to be associated with age and social standing [17]. Surveys of non-human primates have shown that host phylogeny influences the gut eukaryome makeup more than the gut bacteriome composition [19]. The same study showed that the activity of individual hosts and the surrounding environment alter the composition of the gut eukaryome; however, the precise mechanisms were not confirmed [19].

Understanding factors that contribute to the diversity in the gut eukaryome assemblage among hosts is essential, given the influence of the eukaryome on the biological functions of the host. In the scope of this study, we examined the gut eukaryome of two rodent species that inhabit the Jordanian side of the GRV and live under distinct bioclimatic circumstances, *M. musculus* and *A. cahirinus*. Using 18S rRNA gene amplicons, we evaluated the taxonomic diversity, prevalence, and possible pathogenicity of the eukaryome. We then correlated the analysis of the eukaryome taxa with bioclimatic zones. The goal is to advance our knowledge of the diversity patterns of the gut eukaryome and to create a baseline for comparison with future studies.

## Methods

### Study site

**Fig. 1:**
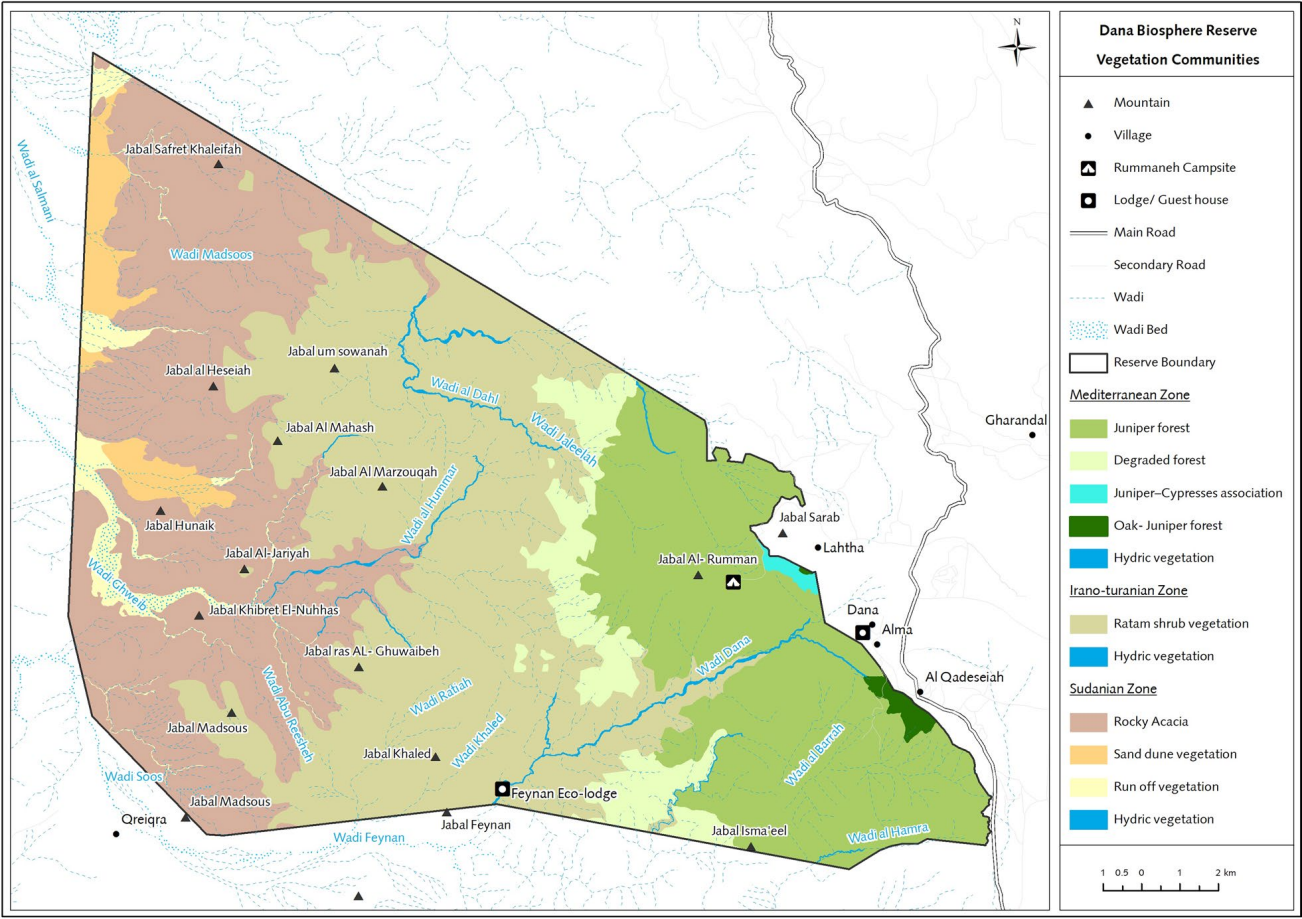
Sampling sites for *Acomys cahirinus* and *Mus musculus domesticus* on the Jordanian side of the Great Rift Valley were located over a short transect crossing a temperate and tropical zone where they coexist

The study covers three different bioclimatic zones, tropical, temperate, and semi-temperate, which are in the GRV and span southern Jordan (Fig. 1). The Mediterranean zone, at heights between 700 and 1,500 m above sea level, facing north toward Europe, contains a semiarid rainforest with a temperate, colder, and moist climate. The Sudanian zone is known for its subtropical Acacia vegetation and receives < 50 mm of rainfall annually. The vegetation in the mid-elevation steppe of the valley, which is located at elevations between 500 and 700 m above sea level, shows signs of an Irano-Turanian ecological environment (Additional file 1: Table S1). Climate research has shown that the Sudanese zone receives up to eight times more solar energy than the Mediterranean region. The average yearly temperature on the Sudanese side is observed to be 10° higher. These elements lead to the development of tropical weather conditions in nearby areas, which are situated near the Dead Sea.

### Rodent sampling and species identification

The comprehensive procedure for collecting rodents and conducting genotyping in this investigation is included in our previously published methodology [27]. Between October 2020 and May 2021, 120 rodents, including *M. mus domesticus* and *A. cahirinus*, were collected from each of the three bioclimatic zones. Sherman traps were placed randomly at the collection site, approximately 10–20 m apart. Animal species were initially identified during trapping based on morphology, as described in Ref. [24]. In addition, we validated the taxonomic categorization and distinguished the rodent species using the *D loop* as a target gene [27]. Upon collection, the weight and reproductive status of each animal were documented. Pregnant females were excluded. The rodents were killed by cervical dislocation promptly following anesthesia. Organs, such as the cecum pouch, were preserved in Zymo’s DNA/RNA Shield, a solution used to maintain biological samples until DNA extraction. The rodents were trapped and treated in accordance with the guidelines of the Jordanian Royal Society for the Conservation of Nature (RSCN).

### PCR amplification and phylogenetic analysis

Genomic DNA was extracted from 200 mg of cecal content material using the QIAamp® Fast DNA Stool Mini Kit following the manufacturer's instructions. The Joint Microbiome Facility of the Medical University of Vienna and the University of Vienna conducted amplicon sequencing and raw data processing utilizing a two-step PCR barcoding method, as outlined in a recent study [28] (project ID JMF-2106-01). The primers used for annealing and amplifying the conserved areas adjacent to the 5′ and 3′ regions of the V4 loop of the 18S rRNA gene were as follows: forward primer (5′-CCAGCASCYGCGGTAATTCC-3′) and reverse primer (5′-ACTTTCGTTCTTGATYRATGA-3′) [29, 30].

We selected primers based on the criteria outlined by Stoek et al. (2010). More than 1000 eukaryotic SSU rDNA sequences were used to construct the primers. This included environmental clone sequences and sequences from all major taxonomic groups [30]. Both primers had a 16-nucleotide head sequence at the 5′ end to enable additional multiplexing. The normalized library was created using adaptor ligation and PCR using the TruSeq Nano DNA Library Prep Kit following the manufacturer's guidelines. It was then sequenced on an Illumina MiSeq platform, v3 2 × 300 bp. Demultiplexing was conducted using the demultiplex Python program by Laros JFJ, available at github.com/jfjlaros/demultiplex, with a tolerance of one mismatch for barcodes and two mismatches for linkers and primers. ASVs were determined using the DADA2 R package version 1.26 [31] by applying the recommended workflow [32]. FASTQ reads 1 and 2 were reduced to 240 nucleotides, with permitted anticipated errors of 4 and 6, respectively. The ASV sequences were categorized using DADA2 against the SILVA 18S database with default parameters [33].

### Statistical analysis

Statistical analyses were conducted using R Studio software (version 4.2.1) (R Core Team, 2022), and visualizations were generated with the ggplot2 package (version 3.4.2). To standardize the number of reads across cecum samples, each sequence count was normalized by the total number of reads in that sample, resulting in relative abundance measures, except for differential expression analysis, where raw sequence counts were utilized. The 18S rRNA libraries underwent rarefaction to a read depth of 100 reads using the rrarefy function within the vegan package (version 2.6.2) [34]. Subsequently, 51 samples were selected for further analysis. Moreover, permutational multivariate analysis of variance (PERMANOVA) was performed using the vegan package to assess the association between gut eukaryome composition and location, host sex, weight, and phylogeny. The identification of indicator ASVs for different zones was conducted utilizing the randomForest package (version 4.7.1.1) [35], and the results were visualized using the ComplexHeatmap package (version 2.15.1). For the random forest analysis, we selected ASVs with a prevalence ≥ 1 and a maximum abundance ≥ 1, resulting in 66 ASVs across 51 mouse samples. To estimate the model’s error rate, we considered Out-Of-Bag (OOB) score and class error rates within the random forest analysis. In addition, taxon enrichment within bioclimatic zones was assessed using the ALDEx2 package (version 1.30.0) [36]. Before the resampling process, the prediction algorithms were used on a group of 51 animals to obtain a full picture of the three bioclimatic zones, excluding any cases where data were missing or of poor quality. This resulted in the inclusion of 25 rodents from the Sudanian zone, 16 from the Mediterranean, and 10 from the Irano-Turanian region (Additional file 2: Table S2).

## Results

### Prevalence and pathogenicity of the eukaryome in the rodent gut

A thorough examination of the intestinal eukaryome of *M. mus domesticus* and *A. cahirinus* was made possible by high-throughput sequencing of multiple 18S rRNA gene amplicons; nonetheless, most of the eukaryotic data were sequence reads from either the host or other gut noninhabitants. After quality screening, a significant number of the data—including those from the taxa Craniata, Arthropoda, Rotifera, and Embryophyceae—were eliminated to reveal the contents of the gut eukaryome (1,038,557 reads were discarded). After filtering, we found a diverse range of eukaryotic gut residents, together with what seem to be fed or accidentally eaten eukaryotes (e.g. plant microbes). Overall, 146,714 quality reads were found, and these were compiled using shared taxonomic assignments, as shown in Additional file 3: Table S3.

Based on the proportionate distribution of assigned reads, helminths (46.5%) and protozoa (45.8%) dominated the eukaryome communities in the rodent gut. Fungi were present in 6.5% of the identified ASVs. Sixty percent (89,118/146,714) were determined to be pathogenic. Based on the information provided in the literature, > 50% of the strains were pathogenic to humans and other animals, 1% (907/89,118) were plant pathogenic species, and the remaining 49% were pathogenic to rodents. The diversity of eukaryote subgroups measured from the gut of the two rodent species as extracted by amplicon sequencing variance (ASVs) of fecal samples are displayed in Table 1.

**Table 1:** The diversity of eukaryote subgroups (Protista, nematodes and fungi) from the gut biome of the two rodent species as determined by analyzing amplicon sequencing variants (ASVs) from fecal samples

Classification	Region			Total
	Irano-Turanian	Mediterranean	Sudanian	
Protists	13	14	11	38
Nematodes	3	3	4	10
Fungi	2	8	7	17
Unidentified*	10	1	2	13

^*^Number of ASVs that are annotated as “undefined” at the phylum level, based on SILVA database

### Eukaryotic organisms in the rodent gut

#### Protista

**Fig. 2:**
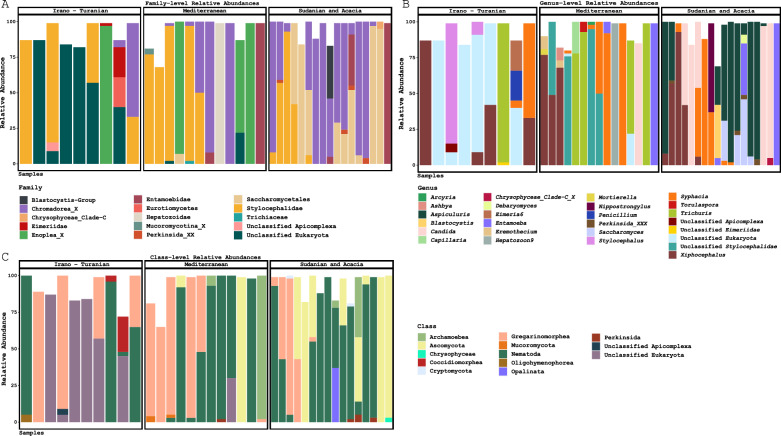
Analysis of the relative abundance of eukaryotic organisms in the cecum of *Acomys cahirinus* and *Mus musculus domesticus* from the three bioclimatic zones. The 18S rRNA gene amplicons were taxonomically classified at the class (**A**), family **B**, and genus (**C**) levels to determine the composition of the fungal, helminth, and protozoan taxa for each sample

We found protists, such as *Entamoeba* sp., that are known to live in the guts of mammals. In addition, there are those that probably enter the rodent's gut because of ingesting insects. Most of the ASVs were from members of the Stylocephalidae family (Fig. 2A), which are often found as symbionts in insects and other invertebrates. These include *Xiphocephalus ellisi* and *Stylocephalus giganteus*. These ASVs are found in 80% of rodents. Among the protists associated with the intestines, *Entamoeba muris* was the most common (Fig. 2B). A low relative number and amount of Blastocystis ASVs were detected. Additional ASVs found included those of Perkinsea and Oligohymenophorea (Fig. 2C). Perkinsea are frequently isolated from fish and bivalve mollusks found in marine environments, while Oligohymenophorea are mostly free living. Since marine food sources are not typically found in mouse diets, we cautiously interpreted the gut eukaryome data. Therefore, the eukaryotic gut signal cannot be separated from the signals of Perkinsea and Oligohymenophorea, whether it is transitional or diet related.

#### Nematodes

All the intestinal helminths that were detected in this study were roundworms. The focus of downstream research was on natural rodent worms because eukaryotic gut signals can be differentiated from the environment or food. The most prevalent taxonomic group, Chromadorea (Fig. 2A), is represented by the pinworm infections (*Oxyurida, Syphacia* and *Syphacia obvelata*) and *Aspiculuris tetraptera*. The latter and the hookworm *Nippostrongylus brasiliensis* were restricted to the tropical bioclimatic zone. On the other hand, ASVs from the Mediterranean region included the whipworms *Enoplea*, *Trichuris vulpis*, and *Capillaria* (Fig. 2B). The latter were also detected in the Irano-Turanian region.

Coinfections are frequent in natural populations. In the present study, we found that 7 out of 50 animals, or 14% of the total, had coinfections with multiple nematode species. Of these, one animal showed double infections with both *A. tetraptera* and *Nippostrongylus*; five showed coinfections with *S. obvelata* and *A. tetraptera*; and one sample each of *Trichuris* sp. and *S. obvelata* or *Capillaria* sp.

#### Fungi

Fungi classified as belonging to all ASVs were found to be present in moderate relative abundance in cecal samples (33%). Ascomycota was the most prevalent phylum of fungi in rodents, especially the subphylum *Saccharomycotina*, which contains *Saccharomyces* and *Candida*. (Fig. 2B). ASVs from the genus *Debaryomyces* and the less common genera *Penicillium*, *Ashbya*, *Torulaspora*, *Clavispora*, and *Eremothecium* were also detected. The main fungal taxa were detected in both rodent species. ASVs belonging to the *Pucciniomycotina* (*Basidiomycota*) and *Mucoromycota* (*Mortierella*) subgroups were found to be less common. These ASVs were detected only in rodents from the Mediterranean zone and are known to be common environmental fungi.

Of the total ASVs in the studied dataset, 16.6% were not classified (Table 1). These ASVs were either not labeled at the subgroup level or annotated with phrases such as "undefined."

 A few ASVs were unclassified at the species level (Fig. 2 B).

### Taxonomic variation in the gut microbiome according to bioclimate zone

The statistical results showed that the gut eukaryome composition significantly differed among the three bioclimatic zones (Table 2). We discovered that location (bioclimate zone) accounted for most of the variation (PERMANOVA, *df* = 2, mean square = 1.64, R^2^ = 0.14, *P* = 0.001). Host species, weight, and sex were not significantly associated with eukaryome composition.

**Table 2:** Permutational multivariate analysis of variance (PERMANOVA) assessing the impact of host characteristics and bioclimatic zones on eukaryotic community composition

Variables	*df*	Mean square	R-square	*P* value
Bioclimatic zone	2	1.64	0.14	0.001
Host sex	1	0.51	0.02	0.265
Host weight	38	0.41	0.71	0.577
Host species	2	0.28	0.02	0.906
Residuals	7	0.42	0.13	–
Total	50	–	1.00	–

*P* < 0.01 indicates significant value

Although some nematodes, such as *S. obvelata*, were cosmopolitan, other phylotypes were bioclimate zone specific. The Sudanian zone had a significantly greater frequency of pinworms than the other bioclimatic zones (Chi-square test, *χ2* = 14.77, *df* = 2, *P* = 0.0006). Most rodents from the Sudanian zone were infected with at least one pinworm species (12/15, or 80%). Whipworms, on the other hand, were found in the gut eukaryomes of rodents that lived in Mediterranean bioclimatic zones, and *Capillaria* was exclusively isolated in this area. Additionally, a greater abundance of *Entamoeba* sp. protozoa was detected in this region (Chi-square test, *χ2* = 3.6, *df* = 1, *P* = 0.05). On the other hand, Stylocephalidae occupied most of the classified sections. In the Iran-Turanian zone, a substantial portion of the eukaryome could not be classified at the genus and species taxonomic levels. However, there was a noticeable *Eimeria*-sourced infection in two of the animals from this location. Here, we observed two main patterns in the eukaryotic gut assemblage of wild rodents: (1) a tendency toward pinworm infection in the Sudanian bioclimatic zone or in Apicomplexa-dominated communities in the Mediterranean zone and (2) considerable variation between individuals within heterospecific rodent species.

**Fig. 3:**
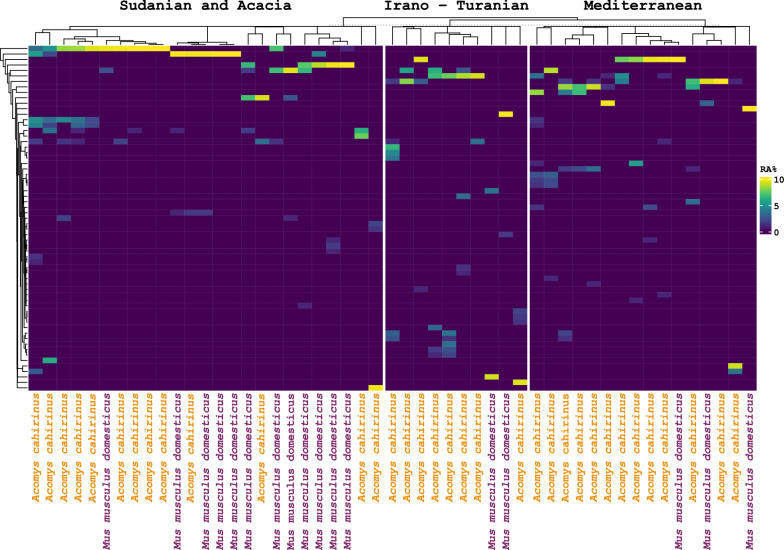
Heatmap of eukaryotic ASV distributions (relative abundance, RA) among the three climate zones according to random forest machine learning (RFML) (n = 51)

We used the random forest machine learning approach (RF-ML) to evaluate the ASVs that were discriminative for the different climate zones in further detail. Only frequent (≥ 1 samples) and abundant (maximum relative abundance ≥ 1%) ASVs, totaling 66, were included in the analysis. This yielded an overall OOB error rate of 17.65%. The model demonstrated high discriminatory power for the Sudanian region, achieving a class error of 0%, indicating perfect classification of Sudanian samples. However, the model's performance was less robust for the Irano-Turanian and Mediterranean regions, with class errors of 70% and 12.5%, respectively. This discrepancy is likely attributable to the small sample sizes and subtle effect sizes distinguishing these environments. Overall, these findings are consistent with the results from the PERMANOVA analysis (Table 2), which also indicated significant differences in the gut microbiome composition among the three regions. The findings show that where host populations live and that their bioclimatic zone may affect the diversity of the gut eukaryome (Fig. 3). In addition, the system revealed 20 ASVs with minimum frequencies of 1% that were specific to the Sudanian bioclimatic zone (Additional file 4: Table S4). This indicates that the rodent eukaryome has a unique makeup that arises from adaptation to the specific bioclimate of the area (Additional files 4–5: Table S4-S5).

## Discussion

In the current investigation, we evaluated the presence of eukaryotic organisms in the cecum of wild rodents *A. cahirinus* and *M. mus domesticus* using 18S rRNA gene metabarcoding. As in other previous studies on eukaryotic metabarcoding [18–20], we amplified a 18S rRNA gene hypervariable region. The three major eukaryotic taxa for which information was collected using this method were fungi, protozoa, and nematodes. This outcome demonstrated the importance of selecting adaptable primers with high coverage for metabarcoding [30]. Targeting multiple markers or using several primer sets simultaneously was used in a few studies, which may have improved the coverage of the eukaryome taxa [17, 37]. Conversely, the advantage of our approach is that it is a robust method with a rapid and uncomplicated laboratory procedure and precise eukaryotic identification, even from composite materials like feces, which have historically proven to be challenging to examine. However, it should be noted that biases inherent in all metabarcoding studies, such as variations in target gene copy number, PCR amplification efficiency, and primer-template mismatches can cause discrepancies between actual relative abundances of organisms in a sample and their relative abundances in sequencing libraries [38]. Therefore, a robust determination of the actual abundances of individual organisms of interest would require suitable alternative methods such as qPCR or microscopic quantification.

The cecum is a unique compartment in the mammalian digestive tract that harbors a microbiota [27]. Cecal flora may be enriched by meals, which also push fluids, substrates, nematodes, and other eukaryotes from the upper gastrointestinal system into the cecum, making it a good site for eukaryome research [39]. In the cecum compartment, we detected eukaryome taxa that are largely consistent with findings from previous research on the eukaryome of the striped field mouse, *Apodemus agrarius* [20].

Because the species that live in the rodent gut must be determined in advance, as well as because of the taxonomic resolution of the data, characterizing the gut eukaryome is challenging. We used variant inference to identify ASVs in mixed populations of eukaryotic species. To do this, we searched the filtered sequences for variants with deep coverage (> 100-fold). Although we achieved a great deal of depth in data acquisition, a portion of the ASVs remains unidentified. This factor certainly affects eukaryome studies, even though it might represent problems with reference sequence databases. Although some approaches, often based on "manual curation," help to alleviate these issues, more efficient methods for identifying and managing eukaryotes accurately are needed. Improvements in longer-read sequencing technology and assembly-based variant calling may be able to address these problems [39].

According to earlier research, the composition of the gut eukaryome varies depending on the host species [40, 41]. This is supported by the comparison between the relative abundance of eukaryome taxa in the wild rodents shown here and the relative abundance of the other species. In the study, the relative abundance of the protozoa and nematodes was greater than that of fungi. This is consistent with findings in the *A. agrarius* [20] but contrasts findings for bats and hyenas [17, 18]. Part of this variation might be attributed to technical reasons. For instance, it is more difficult to extract DNA from fungi and nematodes than from protozoal cells because the former have tough cell walls and the latter have cuticles that are resistant to certain isolation techniques. Additionally, the adult specimens of nematodes have few cells, making it difficult to extract large amounts of genetic material. However, as nematodes are typically expelled in large quantities and certain species hatch prior to feces, live multicellular larvae are present in the cecum section for sequencing.

Rodents are one of several animal species that serve as reservoir hosts for pathogenic eukaryotes [42]. For instance, the zoonotic pathogens *Blastocystis homoinis* and *Trichuris vulpis* are known to cause human diarrhea [43, 44]. Our research also revealed a few possible fungal pathogens, such as *Debaryomyces* sp. and *Candida glabrata*. These opportunistic pathogens can infect people who have diabetes mellitus or immunocompromised individuals [45]. Consequently, profiling of the rodent eukaryome may be valuable for studying events such as the spread of infectious diseases or the creation of new zoonotic infections. Patterns of eukaryotic diversity between bioclimatic zones, as well as within and among host species, might provide valuable insights.

Members of the two rodent species share the primary fungal taxa. Ascomycete fungi that were highly abundant were *Saccharomyces, Candida*, and *Debaryomyces*. These genera were found in both laboratory and wild mouse studies [46, 47], indicating that they are important gut eukaryome species. Our findings are in also line with evidence suggesting that yeasts from the genera *Saccharomyces* and *Candida* dominate the human fungal microbiome [48]. In contrast to this study, the genus *Kazachstania* was highly ubiquitous and numerous in other wild mice, notably *A. agrarius* and *M. musculus* [20, 49].

This study could not differentiate between the protozoa and fungus that are consumed during feeding and those that reside in an animal's intestines. This conclusion about the difficulty of detecting fungi and protozoa in the intestines of wild rats is consistent with that of Kim et al. (2022) [20]. Further investigation into a wide range of wild animal species is necessary to establish the generality of these findings. For omnivorous species, we also need to be able to determine the difference between food source parasites and real host gut eukaryotes.

The nematodes *N. brasiliensis* and *A. tetraptera* were detected in only the Sudanese bioclimatic zone of rodents. *Aspiculuris tetraptera* is commonly isolated from house mice (*M. mus musculus*) [50]. Additionally, other rodent species, such as *Microtus socialis* and *Meriones persicus*, carry it less frequently [51]. The results of the present study, along with those of prior research, suggest that the hookworms *N. brasiliensis* are capable of infecting several species of rodents [52]. However, our results suggest that climate may have an impact on the incidence or distribution of these nematodes. For example, hookworms seek out a host by sensing the warmth of their environment. As a result, their frequency decreases in colder climates [53]. Past studies revealed that parasitic helminths have characteristics that vary with temperature [54]. The distribution of certain parasitic nematodes may differ geographically based on differences in optimal growth conditions between species. The relative predominance of different species might change depending on the location because certain species have growing advantages over others. Our results highlight the significance of considering the potential effects of temperature variations on *A. tetraptera* and *N. brasiliensis* when performing experiments since both species are used as models for many medical nematodes, such as the use of *N. brasiliensis* infection as a murine model of *Necator americanus* [8].

Whipworms including *Trichuris vulpis* and *Capillaria* sp. were found only in the temperate zone. A previous study suggested that climate characteristics, such as precipitation levels, might influence Trichuridae infection in wild rats [55]. A prior study on *Capillaria* spp. revealed a much greater occurrence during the winter season and a negative correlation between the maximum air temperature and degree of infection [56]. Additional studies have demonstrated that *Trichuris* eggs primarily develop between 25 and 26 °C [57]. Whipworm infections and remotely measured mean land surface temperature (LST) are strongly correlated. Whipworms are nearly eliminated in regions where the soil temperature exceeds 38°C [58], and they are most prevalent in areas with LSTs between 25 and 33°C [59]. We examined tropical and temperate bioclimatic zones with temperatures that were adequately within the specified LST restrictions. Given that the average spring and summer temperatures of the Sudanese bioclimatic zone are between 38 and 39°C, our findings that whipworms are absent are consistent with earlier findings that 37 °C is the critical temperature for egg degeneration [60]. Notably, helminth eggs can be found as deep as 10 cm below the soil surface [59], where a number of biotic and abiotic variables shield them from direct sunlight.

Despite the strikingly comparable anatomy between the ova, the diagnosis of trichuroid infestations depends on the identification of the characteristic eggs (e.g. through routine fecal floatation), which presents significant difficulties in epidemiological and clinical contexts. In this study, the concurrent presence of *Capillaria* and *Trichuris vulpis* was effectively accomplished using 18S metabarcoding.

*Syphacia obvelata* was the predominant infectious helminth detected in samples from all bioclimatic zones in our investigation. The present figure is compared to the *S. obvelata* loads reported by Kim et al. (2022), which were found in 25% of the rodents studied [20]. This finding aligns with the widespread occurrence of *S. obvelata* in wild mouse populations [61]. Most coinfections are associated with *S. obvelata*. Although research on the coinfection pattern of nematodes in natural populations is limited, coinfection between *A. tetraptera* and *S. obvelata* is frequently reported, particularly in certain inbred mouse colonies [30].

The eukaryome composition in our study was not significantly impacted by host phylogeny. Individuals belonging to both rodent species often possess a homogeneous eukaryome. This result, along with data showing the influence of bioclimatic zones on the prevalence of protozoa and nematodes, indicates that the host species-level effect may not hold for eukaryome diversity in this study, which could be explained by the independent transitions of parasitism within host taxonomic groups [62]. The fact that *A. cahirinus* and *Mus. musculus* co-exist in the same bioclimatic zone contributed to the trend seen in our survey. The biogeography of the host is a significant determinant that can affect the composition of the rodent gut microbiome, as we have demonstrated in earlier study [27]. These conclusions are also supported by some research that has looked into how the geography affects the eukaryome [63]. Variation in the host diet can frequently be linked to environmentally driven variability in the composition of the gut microbiome [46]. Moreover, it was postulated that diet was the primary cause of the variations in intestinal eukaryome composition between herbivorous and insectivorous bats [18]. In contrast, variations in the makeup of eukaryome communities among various host species were more pronounced than variations in diets among non-human primates [19]. It is probable that the eukaryome compositional variations in the wild mice were brought on by their access to various animal diets at each of the three studied bioclimatic zones, as dietary variables have been demonstrated to affect variations in the eukaryome composition in laboratory mice [64].

We show how bioclimatic conditions influence the diversity of nematode species. Nematodes influence the fitness of their host, potentially impacting the host's ability to adapt to its environment. Although body weight is crucial for a mouse's fitness in the environment [65, 66], we did not find any significant connection between the gut eukaryome and host weight, even when considering nematodes. Conversely, prior studies have demonstrated that intestinal infection significantly affects the weight of laboratory mice [67]. Our findings can provide insight into the capacity of rodents for tolerance. Animal biologists view host tolerance as crucial for wild animals. Host tolerance helps decrease the negative effects of a particular parasite burden on health without eradicating the infection [67, 68]. Studying the relationship between eukaryotic organisms in the intestine and Darwinian fitness can provide insights into how symbiotic organisms in wild mammals contribute to their overall success. Kristan et al. (2004) demonstrated that pinworm infections have a substantial impact on the life cycle of the wild-derived house mouse *M. m. domesticus*, particularly on its reproductive capabilities and the growth of its offspring [69]. Similarly, investigations have shown that *S. obvelata* has the potential to regulate autoimmune disorders [70]. Further research is required to evaluate the fitness costs and benefits associated with gastrointestinal nematodes in wild mammalian species.

## Conclusion

This research revealed a connection between bioclimatic factors and the intestinal eukaryome of small mammals within a natural range. The data could be integrated into models predicting potential risks to ecosystem balance and changes in infection patterns due to global climate change. Future research should focus on expanding sampling efforts to further understand the relationship between host ecology and eukaryome diversity in different bioclimatic zones.

### Supplementary Information

Additional file 1.

Additional file 2.

Additional file 3.

Additional file 4.

Additional file 5.

Additional file 6.

## Data Availability

The datasets generated in this study are available at https://www.ncbi.nlm.nih.gov/, PRJNA992969. Mitochondrial D loop sequences are available in GenBank with accessions ranging from OR613128 to OR613236.

## Abbreviation

GRV: Great Rift Valley

## References

[1] Laforest-Lapointe I, Arrieta MC. Microbial eukaryotes: a missing link in gut microbiome studies. mSystems. 2018;3:e00201-e217. 29556538 10.1128/mSystems.00201-17 PMC5850078

[2] Aivelo T, Medlar A. Opportunities and challenges in metabarcoding approaches for helminth community identification in wild mammals. Parasitology. 2018;145:608–21. 28534454 10.1017/S0031182017000610

[3] Kreisinger J, Bastien G, Hauffe HC, Marchesi J, Perkins SE. Interactions between multiple helminths and the gut microbiota in wild rodents. Philos Trans R Soc B, Biol Sci. 2015;370:20140295. 10.1098/rstb.2014.0295 PMC4528493 26150661

[4] Morton ER, Lynch J, Froment A, Lafosse S, Heyer E, Przeworski M, et al. Variation in rural african gut microbiota is strongly correlated with colonization by entamoeba and subsistence. PLoS Genet. 2015;11:e1005658. 26619199 10.1371/journal.pgen.1005658 PMC4664238

[5] Xu Q, Qiao Q, Gao Y, Hou J, Hu M, Du Y, et al. Gut microbiota and their role in health and metabolic disease of dairy cow. Front Nutr. 2021. 10.3389/fnut.2021.701511. 34422882 10.3389/fnut.2021.701511 PMC8371392

[6] Luo Y, Li J, Zhou H, Yu B, He J, Wu A, et al. The nutritional significance of intestinal fungi: alteration of dietary carbohydrate composition triggers colonic fungal community shifts in a pig model. Appl Environ Microbiol. 2021;87:e00038-e121. 33712429 10.1128/AEM.00038-21 PMC8117771

[7] Sarzhanov F, Dogruman-Al F, Santin M, Maloney JG, Gureser AS, Karasartova D, et al. Investigation of neglected protists *Blastocystis* sp. and *Dientamoeba**fragilis* in immunocompetent and *immunodeficient* diarrheal patients using both conventional and molecular methods. PLoS Negl Trop Dis. 2021. 10.1371/journal.pntd.0009779. 34613993 10.1371/journal.pntd.0009779 PMC8494357

[8] Nair MG, Herbert DR. Immune polarization by hookworms: taking cues from T helper type 2, type 2 innate lymphoid cells and alternatively activated macrophages. Immunology. 2016;148:115–24. 26928141 10.1111/imm.12601 PMC4863575

[9] Brown R, Priest E, Naglik JR, Richardson JP. Fungal toxins and host immune responses. Front Microbiol. 2021;12:643639. 33927703 10.3389/fmicb.2021.643639 PMC8076518

[10] Qamar W, Zaman MA, Faheem M, Ahmed I, Ali K, Qamar MF, et al. Molecular confirmation and genetic characterization of haemonchus contortus isolates at the nuclear ribosomal its2 region: first update from Jhang region of Pakistan. Pak Vet J. 2022;42:251–5.

[11] Gogarten JF, Calvignac-Spencer S, Nunn CL, Ulrich M, Saiepour N, Nielsen HV, et al. Metabarcoding of eukaryotic parasite communities describes diverse parasite assemblages spanning the primate phylogeny. Mol Ecol Resour. 2020;20:204–15. 31600853 10.1111/1755-0998.13101

[12] Tanaka R, Hino A, Tsai IJ, Palomares-Rius JE, Yoshida A, Ogura Y, et al. Assessment of helminth biodiversity in wild rats using 18s rDNA based metagenomics. PLoS ONE. 2014;9:e110769. 25340824 10.1371/journal.pone.0110769 PMC4207705

[13] Aivelo T, Medlar A, Löytynoja A, Laakkonen J, Jernvall J. Tracking year-to-year changes in intestinal nematode communities of rufous mouse lemurs (*Microcebus**rufus*). Parasitology. 2015;142:1095–107. 25892063 10.1017/S0031182015000438

[14] Chihi A, O’Brien Andersen L, Aoun K, Bouratbine A, Stensvold CR. Amplicon-based next-generation sequencing of eukaryotic nuclear ribosomal genes (metabarcoding) for the detection of single-celled parasites in human faecal samples. Parasite Epidemiol Control. 2022;17:e00242. 35146142 10.1016/j.parepi.2022.e00242 PMC8819130

[15] Stensvold CR, Jirků-Pomajbíková K, Tams KW, Jokelainen P, Berg RPKD, Marving E, et al. Parasitic intestinal protists of zoonotic relevance detected in pigs by metabarcoding and real-time PCR. Microorganisms. 2021;9:1189. 34073014 10.3390/microorganisms9061189 PMC8229027

[16] Chen X. Molecular Epidemiological Investigation of *Cryptosporidium* sp., *Giardia**duodenalis*, *Enterocytozoon**bieneusi* and *Blastocystis* sp. infection in free-ranged yaks and tibetan pigs on the plateau. Pak Vet J. 2022;42:533–9. 10.29261/pakvetj/2022.060

[17] Heitlinger E, Ferreira SCM, Thierer D, Hofer H, East ML. The intestinal eukaryotic and bacterial biome of spotted hyenas: the impact of social status and age on diversity and composition. Front Cell Infect Microbiol. 2017;7:262. 28670573 10.3389/fcimb.2017.00262 PMC5472691

[18] Li J, Li L, Jiang H, Yuan L, Zhang L, Ma JE, et al. Fecal bacteriome and mycobiome in bats with diverse diets in south China. Curr Microbiol. 2018;75:1352–61. 29922970 10.1007/s00284-018-1530-0

[19] Mann AE, Mazel F, Lemay MA, Morien E, Billy V, Kowalewski M, et al. Biodiversity of protists and nematodes in the wild nonhuman primate gut. ISME J. 2020;14:609–22. 31719654 10.1038/s41396-019-0551-4 PMC6976604

[20] Kim SL, Choi JH, Myun-Hee Y, Lee S, Kim M, Oh S, et al. Metabarcoding of bacteria and parasites in the gut of *Apodemus**agrarius*. Parasit Vectors. 2022;15:486. 36564849 10.1186/s13071-022-05608-w PMC9789561

[21] Greenspoon L, Krieger E, Sender R, Rosenberg Y, Bar-On YM, Moran U, et al. The global biomass of wild mammals. Proc Natl Acad Sci U S A. 2023. 10.1073/pnas.2204892120. 36848563 10.1073/pnas.2204892120 PMC10013851

[22] Bazzano T, Restel TI, Pinto RM, Gomes DC. Patterns of infection with the nematodes *Syphacia**obvelata* and *Aspiculuris**tetraptera* in conventionally maintained laboratory mice. Mem Inst Oswaldo Cruz. 2002;97:847–53. 12386708 10.1590/S0074-02762002000600017

[23] Yang Z, Grinchuk V, Smith A, Qin B, Bohl JA, Sun R, et al. Parasitic nematode-induced modulation of body weight and associated metabolic dysfunction in mouse models of obesity. Infect Immun. 2013;81:1905–14. 23509143 10.1128/IAI.00053-13 PMC3676010

[24] Amr ZS, Baker MAA, Qumsiyeh M, Eid E. Systematics, distribution and ecological analysis of rodents in Jordan. Zootaxa. 2018;4397:1–94. 29690341 10.11646/zootaxa.4397.1.1

[25] Jiang G, Liu J, Xu L, Yu G, He H, Zhang Z. Climate warming increases biodiversity of small rodents by favoring rare or less abundant species in a grassland ecosystem. Integr Zool. 2013;8:162–74. 23731812 10.1111/1749-4877.12027

[26] Salkeld D, Trivedi M, Schwarzkopf L. Parasite loads are higher in the tropics: temperate to tropical variation in a single host-parasite system. Ecography. 2008;15:538–44. 10.1111/j.0906-7590.2008.05414.x

[27] Al-khlifeh E, Khadem S, Hausmann B, Berry D. Microclimate shapes the phylosymbiosis of rodent gut microbiota in Jordan’s Great Rift Valley. Front microbiol. 2023. 10.3389/fmicb.2023.1258775. 37954239 10.3389/fmicb.2023.1258775 PMC10637782

[28] Pjevac P, Hausmann B, Schwarz J, Kohl G, Herbold CW, Loy A, et al. An economical and flexible dual barcoding, two-step PCR approach for highly multiplexed amplicon sequencing. Front microbiol. 2021. 10.3389/fmicb.2021.669776. 34093488 10.3389/fmicb.2021.669776 PMC8173057

[29] Piredda R, Tomasino MP, D’Erchia AM, Manzari C, Pesole G, Montresor M, et al. Diversity and temporal patterns of planktonic protist assemblages at a mediterranean long term ecological research site. FEMS Microbiol Ecol. 2017. 10.1093/femsec/fiw200. 27677681 10.1093/femsec/fiw200

[30] Stoeck T, Bass D, Nebel M, Christen R, Jones MDM, Breiner HW, et al. Multiple marker parallel tag environmental DNA sequencing reveals a highly complex eukaryotic community in marine anoxic water. Mol Ecol. 2010;19:21–31. 20331767 10.1111/j.1365-294X.2009.04480.x

[31] Callahan BJ, McMurdie PJ, Rosen MJ, Han AW, Johnson AJA, Holmes SP. DADA2: High-resolution sample inference from Illumina amplicon data. Nat Methods. 2016;13:581–3. 27214047 10.1038/nmeth.3869 PMC4927377

[32] Callahan BJ, Sankaran K, Fukuyama JA, McMurdie PJ, Holmes SP. Bioconductor workflow for microbiome data analysis: from raw reads to community analyses. F1000Research. 2016. 10.12688/f1000research.8986.2. 27508062 10.12688/f1000research.8986.2 PMC4955027

[33] Morien E, Parfrey LW. SILVA v128 and v132 dada2 formatted 18s “train sets”. Zenodo; 2018. https://zenodo.org/record/1447330. Accessed 24 Feb 2024.

[34] Oksanen J, Simpson G, Blanchet FG, Kindt R, Legendre P, Minchin P, et al. vegan community ecology package version 2.6–2 April 2022. 2022.

[35] Liaw A, Wiener M. Classification and regression by randomforest. Forest. 2001;30:23.

[36] Gloor GB, Wu JR, Pawlowsky-Glahn V, Egozcue JJ. It’s all relative: analyzing microbiome data as compositions. Ann Epidemiol. 2016;26:322–9. 27143475 10.1016/j.annepidem.2016.03.003

[37] Alberdi A, Aizpurua O, Gilbert M, Bohmann K. Scrutinizing key steps for reliable metabarcoding of environmental samples. Methods Ecol Evol. 2018;9:134–47. 10.1111/2041-210X.12849

[38] Fonseca VG. Pitfalls in relative abundance estimation using eDNA metabarcoding. Mol Ecol Resour. 2018;18:923–6. 10.1111/1755-0998.12902

[39] Hillman ET, Lu H, Yao T, Nakatsu CH. Microbial ecology along the gastrointestinal tract. Microbes Environ. 2017;32:300–13. 29129876 10.1264/jsme2.ME17017 PMC5745014

[40] Ramayo-Caldas Y, Prenafeta-Boldú F, Zingaretti LM, Gonzalez-Rodriguez O, Dalmau A, Quintanilla R, et al. Gut eukaryotic communities in pigs: diversity, composition and host genetics contribution. Anim Microbiome. 2020;2:18. 33499953 10.1186/s42523-020-00038-4 PMC7807704

[41] Arzamani K, Salehi M, Mobedi I, Adinezade A, Hasanpour H, Alavinia M, et al. Intestinal helminths in different species of rodents in north khorasan province. Northeast of Iran Iran J Parasito. 2017;12:267–73. PMC5527038 28761488

[42] Dahmana H, Granjon L, Diagne C, Davoust B, Fenollar F, Mediannikov O. Rodents as hosts of pathogens and related zoonotic disease risk. Pathogens. 2020;9:202. 32164206 10.3390/pathogens9030202 PMC7157691

[43] Sinniah B, Rajeswari B. Blastocystis hominis infection, a cause of human diarrhea. Southeast Asian J Trop Med Public Health. 1994;25:490–3. 7777913

[44] Márquez-Navarro A, García-Bracamontes G, Álvarez-Fernández BE, Ávila-Caballero LP, Santos-Aranda I, Díaz-Chiguer DL, et al. *Trichuris**vulpis* (Froelich, 1789) infection in a child: a case report. Korean J Parasitol. 2012;50:69–71. 22451737 10.3347/kjp.2012.50.1.69 PMC3309054

[45] Desnos-Ollivier M, Ragon M, Robert V, Raoux D, Gantier JC, Dromer F. *Debaryomyces hansenii* (*Candida**famata*), a rare human fungal pathogen often misidentified as pichia guilliermondii (*Candida**guilliermondii*). J Clin Microbiol. 2008;46:3237–42. 18701668 10.1128/JCM.01451-08 PMC2566122

[46] Mims TS, Abdallah QA, Stewart JD, Watts SP, White CT, Rousselle TV, et al. The gut mycobiome of healthy mice is shaped by the environment and correlates with metabolic outcomes in response to diet. Commun Biol. 2021;4:1–11. 33674757 10.1038/s42003-021-01820-z PMC7935979

[47] Heisel T, Montassier E, Johnson A, Al-Ghalith G, Lin YW, Wei LN, et al. High-fat diet changes fungal microbiomes and interkingdom relationships in the murine gut. mSphere. 2017. 10.1128/msphere.00351-17. 29034327 10.1128/msphere.00351-17 PMC5636226

[48] Pérez JC. Fungi of the human gut microbiota: roles and significance. Int J Med Microbiol. 2021;311:151490. 33676239 10.1016/j.ijmm.2021.151490

[49] Bendová B, Piálek J, Ďureje Ľ, Schmiedová L, Čížková D, Martin JF, et al. How being synanthropic affects the gut bacteriome and mycobiome: comparison of two mouse species with contrasting ecologies. BMC Microbiol. 2020;20:194. 32631223 10.1186/s12866-020-01859-8 PMC7336484

[50] Behnke JM, Stewart A, Bajer A, Grzybek M, Harris PD, Lowe A, et al. Bank voles (*Myodes**glareolus*) and house mice (*Mus**musculus**musculus*; *M*. *m*. *domesticus*) in Europe are each parasitized by their own distinct species of *Aspiculuris* (Nematoda, Oxyurida). Parasitology. 2015;142:1493–505. 26302680 10.1017/S0031182015000864

[51] Kia E, Shahryary-Rad E, Mohebali M, Mahmoudi M, Mobedi I, Zahabiun F, et al. Endoparasites of rodents and their zoonotic importance in germi, dashte-mogan, ardabil province. Iran Iran J Parasit. 2010;5:15–20. PMC3279854 22347261

[52] Montaño KJ, Cuéllar C, Sotillo J. Rodent models for the study of soil-transmitted helminths: a proteomics approach. Front Cell Infect Microbiol. 2021. 10.3389/fcimb.2021.639573. 33968800 10.3389/fcimb.2021.639573 PMC8100317

[53] Mabaso MLH, Appleton CC, Hughes JC, Gouws E. The effect of soil type and climate on hookworm (*Necator**americanus*) distribution in KwaZulu-Natal. South Africa Trop Med Int Health. 2003;8:722–7. 12869093 10.1046/j.1365-3156.2003.01086.x

[54] Phillips JA, Vargas Soto JS, Pawar S, Koprivnikar J, Benesh DP, Molnár PK. The effects of phylogeny, habitat and host characteristics on the thermal sensitivity of helminth development. Proc R Soc B. 2022;289:20211878. 35135354 10.1098/rspb.2021.1878 PMC8825990

[55] Thomas C, Msoffe V, Van Houtte N, Mhamphi G, Mariën J, Sabuni C, et al. Prevalence and seasonal variation of *Trichuris* worms infection in *mastomys**natalensis* in morogoro and iringa regions. Tanzania Parasitologia. 2023;3:293–9. 10.3390/parasitologia3030030

[56] Pilarczyk B, Tomza-Marciniak A, Pilarczyk R, Sadowska N, Udała J, Kuba J. The effect of season and meteorological conditions on parasite infection in farm-maintained mouflons (*Ovis**aries**Musimon*). J Parasitol Res. 2022;2022:1165782. 35127154 10.1155/2022/1165782 PMC8808193

[57] Forman R, Partridge FA, Sattelle DB, Else KJ. Un-‘Egg’-Plored: characterisation of embryonation in the whipworm model organism *Trichuris**muris*. Front Trop Dis. 2021. 10.3389/fitd.2021.790311. 10.3389/fitd.2021.790311

[58] Fahmy MA. An investigation on the life cycle of *Trichuris**muris*. Parasitology. 1954;44:50–7. 13166371 10.1017/S003118200001876X

[59] Brooker S, Clements ACA, Bundy DAP. Global epidemiology, ecology and control of soil-transmitted helminth infections. In: Hay SI, Graham A, Rogers DJ, editors. Int J Parasitol Parasites. Academic Press; 2006. p. 221–61. 10.1016/S0065-308X(05)62007-6 PMC1976253 16647972

[60] Paller VGV, de Chavez ERC. *Toxocara* (Nematoda: Ascaridida) and other soil-transmitted helminth eggs contaminating soils in selected urban and rural areas in the Philippines. Sci World J. 2014;2014:386232. 10.1155/2014/386232 PMC4212593 25383372

[61] Islam MM, Farag E, Hassan MM, Bansal D, Awaidy SA, Abubakar A, et al. Helminth parasites among rodents in the middle east countries: a systematic review and meta-analysis. Animals. 2020;10:2342. 33317021 10.3390/ani10122342 PMC7764038

[62] Weinstein SB, Kuris AM. Independent origins of parasitism in Animalia. Biol Lett. 2016;12:20160324. 27436119 10.1098/rsbl.2016.0324 PMC4971171

[63] Vonaesch P, Billy V, Mann AE, Morien E, Habib A, Collard JM, et al. The eukaryome of African children is influenced by geographic location, gut biogeography, and nutritional status. Microlife. 2023. 10.1093/femsml/uqad033. 37680753 10.1093/femsml/uqad033 PMC10481997

[64] Gupta Y, Ernst AL, Vorobyev A, Beltsiou F, Zillikens D, Bieber K, et al. Impact of diet and host genetics on the murine intestinal mycobiome. Nat Commun. 2023;14:834. 36788222 10.1038/s41467-023-36479-z PMC9929102

[65] Schulte-Hostedde A, Millar J, Hickling G. Evaluating body condition in small mammals. Can J Zool. 2001;1:1021–9. 10.1139/z01-073

[66] Al-khlifeh E, Alshammari A, Alnasarat H. High incidence of G1 genotype found in the levant revealed by sequence-based association analysis of Echinococcus granulosus (sensu stricto). Pak Vet J. 2024;44(2):405–13.

[67] Råberg L, Sim D, Read AF. Disentangling genetic variation for resistance and tolerance to infectious diseases in animals. Science. 2007;318:812–4. 17975068 10.1126/science.1148526

[68] de Oliveira Terceiro FE, de Arruda MF, Van Schaik CP, Araújo A, Burkart JM. Higher social tolerance in wild versus captive common marmosets: the role of interdependence. Sci Rep. 2021;11:825. 33436898 10.1038/s41598-020-80632-3 PMC7804027

[69] Kristan DM. Intestinal nematode infection affects host life history and offspring susceptibility to parasitism. J Anim Ecol. 2004;73:227–38. 10.1111/j.0021-8790.2004.00794.x

[70] Taghipour N, Mosaffa N, Aghdaei HA, Rostami-Nejad M, Weinstock JV, Shahnavaz S, et al. Immunomodulatory effect of *Syphacia**obvelata* in treatment of experimental DSS-induced colitis in mouse model. Sci Rep. 2019;9:19127. 31836772 10.1038/s41598-019-55552-6 PMC6911064

