# Supplementary material for: Climate influences the gut eukaryome of wild rodents in the Great Rift Valley of Jordan
Source: Parasit Vectors. 2024 Aug 23;17:358. doi: 10.1186/s13071-024-06451-x (PMC11342738; doi:10.1186/s13071-024-06451-x)
Supplement: Supplementary file 1 — Additional file 1. [file 13071_2024_6451_MOESM1_ESM.docx]

**Additional file 1: Table S1.** Environmental conditions and soil properties specific to each zone

| Bioclimatic zone | | | |
| --- | --- | --- | --- |
|  | Mediterranean | Irano-Turanian | Sudanian |
| Altitude (m above sea level) | above c. 700 | 400-700 | 400 to -400 m (below sea level) |
| Max. Annual mean temperature (°C) | 15-20°C | 15-25°C | 20-35°C |
| Min. Annual mean temperature (°C) | 5-10°C | 2‑5°C | 10-20°C |
| Annual Precipitation (mm) | 300 to 600 mm | 150 to 300 mm | 50-100 mm |
| Soil texture | Terra Rossa (Limestone) and yellow Mediterranean soil (Rendzina) | Calcareous and transported by wind | Alluvial transported by water, saline, sandy and granite |
