# Supplementary material for: Climate influences the gut eukaryome of wild rodents in the Great Rift Valley of Jordan
Source: Parasit Vectors. 2024 Aug 23;17:358. doi: 10.1186/s13071-024-06451-x (PMC11342738; doi:10.1186/s13071-024-06451-x)
Supplement: Supplementary file 2 — Additional file 2. [file 13071_2024_6451_MOESM2_ESM.docx]

**Additional file 2: Table S2.** Features of the studied wild rodents. These classifications are based on the samples’ morphology. We also identified the samples based on D-loop sequencing, however, due to insufficient or loos of the sequences, we couldn’t confirm them all (highlighted samples).

| Bioclimatic zone | Rodent species | Sample Code | Body weight | Sex |
| --- | --- | --- | --- | --- |
| Sudanian | *Mus musculus domesticus* | JMF-2106-01-0043 | 17 | Female |
|  | *Mus musculus domesticus* | JMF-2106-01-0044 | 19 | Male |
|  | *Mus musculus domesticus* | JMF-2106-01-0045 | 21 | Female |
|  | *Mus musculus domesticus* | JMF-2106-01-0046 | 17 | Male |
|  | *Mus musculus domesticus* | JMF-2106-01-0047 | 18 | Female |
|  | *Acomys cahirinus* | JMF-2106-01-0050 | 19 | Male |
|  | *Mus musculus domesticus* | JMF-2106-01-0052 | 16 | Female |
|  | *Mus musculus domesticus* | JMF-2106-01-0053 | 18 | Female |
|  | *Mus musculus domesticus* | JMF-2106-01-0054 | 20 | Female |
|  | *Mus musculus domesticus* | JMF-2106-01-0058 | 15 | Female |
|  | *Acomys cahirinus* | JMF-2106-01-0061 | 22 | Female |
|  | *Acomys cahirinus* | JMF-2106-01-0087 | 17.5 | Female |
|  | *Acomys cahirinus* | JMF-2106-01-0089 | 15.5 | Male |
|  | *Acomys cahirinus* | JMF-2106-01-0090 | 14.7 | Male |
|  | *Acomys cahirinus* | JMF-2106-01-0092 | 16.5 | Female |
|  | *Acomys cahirinus* | JMF-2106-01-0093 | 14.1 | Female |
|  | *Acomys cahirinus* | JMF-2106-01-0095 | 11.4 | Male |
|  | *Acomys cahirinus* | JMF-2106-01-0096 | 11.2 | Male |
|  | *Acomys cahirinus* | JMF-2106-01-0097 | 11.2 | Male |
|  | *Acomys cahirinus* | JMF-2106-01-0098 | 15.1 | Male |
|  | *Acomys cahirinus* | JMF-2106-01-0099 | 16.5 | Female |
|  | *Acomys cahirinus* | JMF-2106-01-0100 | 11.5 | Male |
|  | *Mus musculus domesticus* | JMF-2106-01-0101 | 18.7 | Female |
|  | *Mus musculus domesticus* | JMF-2106-01-0103 | 16.5 | Male |
|  | *Mus musculus domesticus* | JMF-2106-01-0104 | 11.5 | Male |
| Mediterranean | *Acomys cahirinus* | JMF-2106-01-0001 | 41 | Male |
|  | *Acomys cahirinus* | JMF-2106-01-0003 | 34 | Male |
|  | *Acomys cahirinus* | JMF-2106-01-0004 | 44 | Male |
|  | *Acomys cahirinus* | JMF-2106-01-0005 | 31 | Male |
|  | *Acomys cahirinus* | JMF-2106-01-0006 | 35 | Female |
|  | *Acomys cahirinus* | JMF-2106-01-0007 | 34 | Female |
|  | *Acomys cahirinus* | JMF-2106-01-0009 | 35 | Male |
|  | *Acomys cahirinus* | JMF-2106-01-0012 | 40 | Female |
|  | *Mus musculus domesticus* | JMF-2106-01-0013 | 52 | Female |
|  | *Mus musculus domesticus* | JMF-2106-01-0015 | 33 | Male |
|  | *Mus musculus domesticus* | JMF-2106-01-0017 | 60 | Male |
|  | *Acomys cahirinus* | JMF-2106-01-0066 | 30 | Female |
|  | *Acomys cahirinus* | JMF-2106-01-0067 | 33 | Female |
|  | *Acomys cahirinus* | JMF-2106-01-0069 | 30 | Female |
|  | *Acomys cahirinus* | JMF-2106-01-0073 | 32 | Female |
|  | *Acomys cahirinus* | JMF-2106-01-0080 | 22 | Male |
| Irano-Turanian | *Mus musculus domesticus* | JMF-2106-01-0028 | 37 | Female |
|  | *Mus musculus domesticus* | JMF-2106-01-0029 | 39 | Male |
|  | *Acomys cahirinus* | JMF-2106-01-0031 | 42 | Female |
|  | *Acomys cahirinus* | JMF-2106-01-0032 | 38 | Male |
|  | *Acomys cahirinus* | JMF-2106-01-0033 | 39 | Female |
|  | *Acomys cahirinus* | JMF-2106-01-0037 | 38 | Male |
|  | *Acomys cahirinus* | JMF-2106-01-0039 | 48 | Male |
|  | *Acomys cahirinus* | JMF-2106-01-0040 | 39 | Male |
|  | *Acomys cahirinus* | JMF-2106-01-0117 | 38.31 | Male |
|  | *Acomys cahirinus* | JMF-2106-01-0118 | 65.5 | Female |
