# Supplementary material for: Climate influences the gut eukaryome of wild rodents in the Great Rift Valley of Jordan
Source: Parasit Vectors. 2024 Aug 23;17:358. doi: 10.1186/s13071-024-06451-x (PMC11342738; doi:10.1186/s13071-024-06451-x)
Supplement: Supplementary file 3 — Additional file 3. [file 13071_2024_6451_MOESM3_ESM.docx]

**Additional file 3: Table S3.** Taxonomic assignments and pathogenicity of the gut eukaryotes found in *M. mus domesticus* and *A. cahirinus*

| Kingdom | | | | | Pathogenicity | | | Part of microbial composition | | | |
| --- | --- | --- | --- | --- | --- | --- | --- | --- | --- | --- | --- |
|  | Animalia | Fungi | Protista | Unknown | Rodents | Animals and Human | Plants | Rodents | Animals and human | Plants | Environment |
| Number of reads | 68316 | 9385 | 67150 | 1863 | 40829 | 47382 | 907 | 69471 | 31895 | 4 | 105 |
| Total | 146714 | | | | 89118 | | | 101475 | | | |
