# Supplementary material for: Climate influences the gut eukaryome of wild rodents in the Great Rift Valley of Jordan
Source: Parasit Vectors. 2024 Aug 23;17:358. doi: 10.1186/s13071-024-06451-x (PMC11342738; doi:10.1186/s13071-024-06451-x)
Supplement: Supplementary file 4 — Additional file 4. [file 13071_2024_6451_MOESM4_ESM.docx]

**Additional file 4: Table S4.** Unique ASVs in Sudanian bioclimatic zone

| Sequence_ID | Host | Class | Order | Family | Genus | Species |
| --- | --- | --- | --- | --- | --- | --- |
| ASV_1km_gh8 | *Acomys.cahirinus* & Mus.musculus.domesticus | Archamoebea | Archamoebea_X | Entamoebidae | *Entamoeba* | *Entamoeba_muris* |
| ASV_1px_da2 | *A.cahirinus* & *M. m. domesticus* | Nematoda | Chromadorea | Chromadorea_X | *Aspiculuris* | *Aspiculuris_tetraptera* |
| ASV_2c8_arm | *A.cahirinus* & *M. m. domesticus* | Cryptomycota | Cryptomycotina | Cryptomycotina_X | *Cryptomycotina_XX* | *Cryptomycotina_XX_sp.* |
| ASV_2nd_w2t | *A.cahirinus* | Opalinata | Opalinata_X | Blastocystis-Group | *Blastocystis* | *Blastocystis_hominis* |
| ASV_3yy_8jm | *M. m. domesticus* | Ascomycota | Saccharomycotina | Saccharomycetales | *Clavispora* | Unclassified *Clavispora* |
| ASV_457_tsw | *A.cahirinus* | Opalinata | Opalinata_X | Blastocystis-Group | *Blastocystis* | *Blastocystis_hominis* |
| ASV_65z_hj7 | *A.cahirinus* | Opalinata | Opalinata_X | Blastocystis-Group | *Blastocystis* | *Blastocystis_hominis* |
| ASV_6a4_rmi | *A.cahirinus* & *M. m. domesticus* | Unclassified | Unclassified | Unclassified | Unclassified | Unclassified *Eukaryota* |
| ASV_6bq_xwj | *M. m. domesticus* & Unclassified host | Archamoebea | Archamoebea_X | Entamoebidae | *Entamoeba* | *Entamoeba_muris* |
| ASV_6kd_nqe | *A.cahirinus* | Ascomycota | Saccharomycotina | Saccharomycetales | *Debaryomyces* | *Debaryomyces_hansenii* |
| ASV_a2x_tai | *A.cahirinus* | Ascomycota | Saccharomycotina | Saccharomycetales | *Saccharomyces* | *Saccharomyces_cerevisiae* |
| ASV_b1y_7h1 | *M. m. domesticus* & Unclassified host | Nematoda | Chromadorea | Chromadorea_X | *Syphacia* | *Syphacia_obvelata* |
| ASV_csm_keu | *M. m. domesticus* | Unclassified | Unclassified | Unclassified | Unclassified | Unclassified *Eukaryota* |
| ASV_ewl_ujv | *A.cahirinus* | Heterotrichea | Heterotrichea_X | Stentoridae | *Stentor* | *Stentor_roeselii* |
| ASV_g4v_3mx | *A.cahirinus* , *M. m. domesticus* & Unclassified host | Nematoda | Chromadorea | Chromadorea_X | *Syphacia* | *Syphacia_obvelata* |
| ASV_j70_oht | *M. m. domesticus* | Ascomycota | Saccharomycotina | Saccharomycetales | *Candida* | *Candida_glabrata* |
| ASV_jih_h11 | *A.cahirinus* | Nematoda | Chromadorea | Chromadorea_X | *Nippostrongylus* | *Nippostrongylus_brasiliensis* |
| ASV_m83_mth | *M. m. domesticus* | Chrysophyceae | Chrysophyceae_X | Chrysophyceae_Clade-C | *Chrysophyceae_Clade-C_X* | *Chrysophyceae_Clade-C_X_sp.* |
| ASV_n7q_v82 | *M. m. domesticus* | Gregarinomorphea | Neogregarinorida | Stylocephalidae | *Xiphocephalus* | Unclassified *Xiphocephalus* |
| ASV_ojr_imt | Unclassified host | Opalinata | Opalinata_X | Blastocystis-Group | *Blastocystis* | *Blastocystis_sp.* |
