# Supplementary material for: Climate influences the gut eukaryome of wild rodents in the Great Rift Valley of Jordan
Source: Parasit Vectors. 2024 Aug 23;17:358. doi: 10.1186/s13071-024-06451-x (PMC11342738; doi:10.1186/s13071-024-06451-x)
Supplement: Supplementary file 5 — Additional file 5. [file 13071_2024_6451_MOESM5_ESM.docx]

**Additional file 5: Table S5.** Unique ASVs in Irano-Turanian bioclimatic zone

| Sequence_ID | Host | Class | Order | Family | Genus | Species |
| --- | --- | --- | --- | --- | --- | --- |
| ASV_3pv_trv | *Acomys cahirinus* | Unclassified Eukaryota | Unclassified Eukaryota | Unclassified Eukaryota | Unclassified *Eukaryota* | Unclassified *Eukaryota* |
| ASV_6aj_eme | *A. cahirinus* | Unclassified Eukaryota | Unclassified Eukaryota | Unclassified Eukaryota | Unclassified *Eukaryota* | Unclassified *Eukaryota* |
| ASV_8it_23u | *A. cahirinus* | Ascomycota | Pezizomycotina | Eurotiomycetes | *Penicillium* | Unclassified *Penicillium* |
| ASV_9an_gmx | *A. cahirinus* | Unclassified Eukaryota | Unclassified Eukaryota | Unclassified Eukaryota | Unclassified *Eukaryota* | Unclassified *Eukaryota* |
| ASV_9e7_qph | *A. cahirinus* | Gregarinomorphea | Neogregarinorida | Actinocephalidae | *Syncystis* | *Syncystis_mirabilis* |
| ASV_b2o_hj7 | *Mus musculus domesticus* | Unclassified Eukaryota | Unclassified Eukaryota | Unclassified Eukaryota | Unclassified *Eukaryota* | Unclassified *Eukaryota* |
| ASV_bg9_qfo | *A. cahirinus* | Unclassified Eukaryota | Unclassified Eukaryota | Unclassified Eukaryota | Unclassified *Eukaryota* | Unclassified *Eukaryota* |
| ASV_dbq_red | *A. cahirinus* | Unclassified Eukaryota | Unclassified Eukaryota | Unclassified Eukaryota | Unclassified *Eukaryota* | Unclassified *Eukaryota* |
| ASV_e3m_jbl | *A. cahirinus* & *M. m. domesticus* | Oligohymenophorea | Hymenostomatia | Ophryoglenida | *Ichthyophthirius* | *Ichthyophthirius_multifiliis* |
| ASV_gcv_kd8 | *A. cahirinus* | Coccidiomorphea | Eimeriida | Eimeriidae | Unclassified *Eimeriidae* | Unclassified *Eimeriidae* |
| ASV_gz4_pk7 | *M. m. domesticus* | Gregarinomorphea | Neogregarinorida | Stylocephalidae | *Xiphocephalus* | *Xiphocephalus_ellisi* |
| ASV_i56_ao0 | *A. cahirinus* | Gregarinomorphea | Neogregarinorida | Stylocephalidae | *Stylocephalus* | *Stylocephalus_giganteus* |
| ASV_k1x_vga | *A. cahirinus* | Unclassified Eukaryota | Unclassified Eukaryota | Unclassified Eukaryota | Unclassified *Eukaryota* | Unclassified *Eukaryota* |
| ASV_k9j_1pn | *M. m. domesticus* | Nematoda | Chromadorea | Chromadorea_X | *Dentostomella* | *Dentostomella_sp.* |
| ASV_kyu_lxp | *A. cahirinus* | Unclassified Eukaryota | Unclassified Eukaryota | Unclassified Eukaryota | Unclassified *Eukaryota* | Unclassified *Eukaryota* |
| ASV_o2r_lzx | *A. cahirinus* | Coccidiomorphea | Eimeriida | Eimeriidae | *Eimeria6* | *Eimeria6_sp.* |
| ASV_ojc_1fd | *A. cahirinus* | Unclassified Apicomplexa | Unclassified Apicomplexa | Unclassified Apicomplexa | Unclassified *Apicomplexa* | Unclassified *Apicomplexa* |
| ASV_oy7_btw | *A. cahirinus* | Ascomycota | Saccharomycotina | Saccharomycetales | *Priceomyces* | Unclassified *Priceomyces* |
| ASV_pfm_yim | *A. cahirinus* | Unclassified Eukaryota | Unclassified Eukaryota | Unclassified Eukaryota | Unclassified *Eukaryota* | Unclassified *Eukaryota* |
| ASV_rxd_41r | *A. cahirinus* | Gregarinomorphea | Neogregarinorida | Stylocephalidae | *Stylocephalus* | *Stylocephalus_giganteus* |
