# Supplementary material for: Climate influences the gut eukaryome of wild rodents in the Great Rift Valley of Jordan
Source: Parasit Vectors. 2024 Aug 23;17:358. doi: 10.1186/s13071-024-06451-x (PMC11342738; doi:10.1186/s13071-024-06451-x)
Supplement: Supplementary file 6 — Additional file 6. [file 13071_2024_6451_MOESM6_ESM.docx]

**Additional file 6: Table S6.** Unique ASVs in Mediterranean bioclimatic zone

| Sequence_ID | Host | Class | Order | Family | Genus | Species |
| --- | --- | --- | --- | --- | --- | --- |
| ASV_1ix_801 | *Acomys cahirinus* | Ascomycota | Saccharomycotina | Saccharomycetales | *Ashbya* | *Ashbya_gossypii* |
| ASV_1wl_10e | *A. cahirinus* | Ascomycota | Saccharomycotina | Saccharomycetales | *Eremothecium* | Unclassified *Eremothecium* |
| ASV_2cs_lsy | Unclassified host | Gregarinomorphea | Neogregarinorida | Stylocephalidae | Unclassified *Stylocephalidae* | Unclassified *Stylocephalidae* |
| ASV_48c_ily | *A. cahirinus* | Oligohymenophorea | Peritrichia_2 | Sessilida | *Vorticella* | *Vorticella_campanula* |
| ASV_bhe_auy | *A. cahirinus* & *Mus musculus domesticus* | Coccidiomorphea | Adeleida | Hepatozoidae | *Hepatozoon9* | *Hepatozoon9_ayorgbor* |
| ASV_ddc_gyn | *A. cahirinus* & *M. m. domesticus* | Archamoebea | Archamoebea_X | Entamoebidae | *Entamoeba* | *Entamoeba_muris* |
| ASV_dks_9st | *A. cahirinus* & *M. m. domesticus* | Coccidiomorphea | Eimeriida | Eimeriidae | Unclassified *Eimeriidae* | Unclassified *Eimeriidae* |
| ASV_fld_rsp | *A. cahirinus* | Ascomycota | Saccharomycotina | Saccharomycetales | *Candida* | *Candida_saitoana* |
| ASV_gik_mfq | *A. cahirinus* | Mycetozoa-Myxogastrea | Trichiales | Trichiaceae | *Arcyria* | *Arcyria_cinerea* |
| ASV_h06_ru1 | *A. cahirinus* | Nematoda | Enoplea | Enoplea_X | *Capillaria* | *Capillaria_xenopi* |
| ASV_i72_qn4 | *A. cahirinus* | Dinophyceae | Unclassified Dinophyceae | Unclassified Dinophyceae | Unclassified *Dinophyceae* | Unclassified *Dinophyceae* |
| ASV_mpp_m60 | *A. cahirinus* | Basidiomycota | Pucciniomycotina | Microbotryomycetes | *Microbotryomycetes_X* | *Microbotryomycetes_X_sp.* |
| ASV_njt_bh9 | *A. cahirinus* | Mucoromycota | Mucoromycotina | Mucoromycotina_X | *Mortierella* | *Mortierella_hyalina* |
| ASV_r6m_xj6 | *A. cahirinus* & Unclassified host | Gregarinomorphea | Neogregarinorida | Stylocephalidae | Unclassified *Stylocephalidae* | Unclassified *Stylocephalidae* |
| ASV_rz2_hs3 | *A. cahirinus* | Ascomycota | Saccharomycotina | Saccharomycetales | *Torulaspora* | *Torulaspora_delbrueckii* |
| ASV_sf6_k7j | *A. cahirinus* | Gregarinomorphea | Neogregarinorida | Stylocephalidae | *Xiphocephalus* | *Xiphocephalus_ellisi* |
